# Near-Complete Genome Sequencing of Swine Vesicular Disease Virus Using the Roche GS FLX Sequencing Platform

**DOI:** 10.1371/journal.pone.0097180

**Published:** 2014-05-09

**Authors:** Sandra C. Abel Nielsen, Christian A. W. Bruhn, Jose Alfredo Samaniego, Jemma Wadsworth, Nick J. Knowles, M. Thomas P. Gilbert

**Affiliations:** 1 Centre for GeoGenetics, Natural History Museum of Denmark, University of Copenhagen, Copenhagen, Denmark; 2 Vesicular Disease Reference Laboratory Group, The Pirbright Institute, Pirbright, Surrey, United Kingdom; The University of Hong Kong, Hong Kong

## Abstract

Swine vesicular disease virus (SVDV) is an enterovirus that is both genetically and antigenically closely related to human coxsackievirus B5 within the *Picornaviridae* family. SVDV is the causative agent of a highly contagious (though rarely fatal) vesicular disease in pigs. We report a rapid method that is suitable for sequencing the complete protein-encoding sequences of SVDV isolates in which the RNA is relatively intact. The approach couples a single PCR amplification reaction, using only a single PCR primer set to amplify the near-complete SVDV genome, with deep-sequencing using a small fraction of the capacity of a Roche GS FLX sequencing platform. Sequences were initially verified through one of two criteria; either a match between a *de novo* assembly and a reference mapping, or a match between all of five different reference mappings performed against a fixed set of starting reference genomes with significant genetic distances within the same species of viruses. All reference mappings used an iterative method to avoid bias. Further verification was achieved through phylogenetic analysis against published SVDV genomes and additional *Enterovirus B* sequences. This approach allows high confidence in the obtained consensus sequences, as well as provides sufficiently high and evenly dispersed sequence coverage to allow future studies of intra-host variation.

## Introduction

Swine vesicular disease (SVD) is a highly contagious viral disease of pigs. The first outbreak of SVD was observed in 1966 in Italy, although at the time, it was misidentified as foot-and-mouth disease (FMD) [1]. Shortly after, the causative agent, SVD virus (SVDV), was shown to be an enterovirus [1]–[3] and classified as a member of the *Enterovirus B* species within the *Picornaviridae* family. It is a non-enveloped virus with a single-stranded RNA genome of positive polarity of approximately 7.4 kb in length [4], [5], and shares the same physico-chemical properties of other enteroviruses [1], [3]. Previous studies have confirmed that SVDV is antigenically related to the human pathogen coxsackievirus B5 (CV-B5) [6], [7], leading Graves to propose that SVD and its causative agent most likely originated from infection of pigs with a human CV-B5. A study comparing the complete nucleotide sequence of two SVDVs and one CV-B5, reported that CV-B5 is more closely related to SVDV than to other coxsackie B viruses in the regions encoding the major capsid proteins 1B, 1C and 1D (also known as VP2, VP3 and VP1, respectively) [8]. Another study by Zhang *et al.* [9] investigated the molecular evolution of SVDV. Here, they analyzed sequence data from 42 SVDV isolates and seven CV-B5 isolates in two shorter regions of the viral genomes; the regions encoding the capsid protein VP1 and the non-structural 3BC corresponding to 283 and 205 amino acids, respectively. They showed that SVDV formed a single monophyletic group, which was clearly distinct from any included CV-B5 as well as other coxsackieviruses included in the study. Furthermore, for each of these regions, the date for which SVDV is believed to have originated in pigs was estimated to have been between 1945 and 1965.

**Figure 1:**
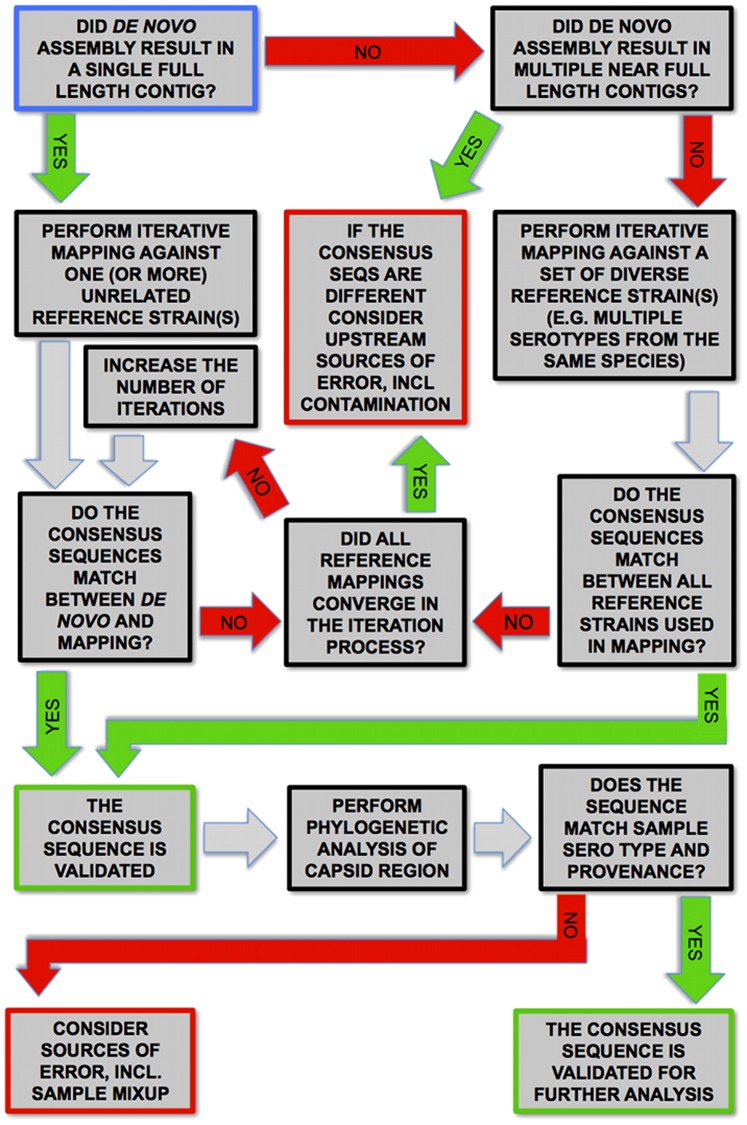
Decision diagram for validation of consensus sequences. The diagram illustrates the logic of the applied methodology for obtaining consensus sequences validated for further analysis. The process begins with *de novo* assembly as described in the methods section. The starting point from there is the blue-outline box in the top left hand corner. Positive answers follow the green ‘YES’ arrows, negative ones follow the red ‘NO’ arrows, and grey arrows are followed in all cases. Termination in a red box should lead to thorough analysis of upstream sources of error, including everything from contaminated samples to late stage *in silico* problems. Arriving at the first green box means that the consensus sequence of assembly/mapping has been verified. Arriving at the final green box means that the sample sequence is fully validated, now also with regard to sample provenance. The sample sequence is now ready to be used for further scientific analysis.

Due to improvements in DNA sequencing technologies [10], and hence the ease with which sequencing data can be generated, sequencing and characterization of viral genomes has accelerated. Because SVDV is of ongoing veterinary concern, we anticipate future studies will have an interest in obtaining increased numbers of SVDV coding-region sequences. Therefore, we developed a simple method to reconstruct near-complete SVDV genomes (encompassing the complete coding region) that is suitable for application to SVDV isolates in which RNA is largely intact. The method is based on PCR amplification using only a single primer set to generate the near-complete SVDV genome, which is performed after first-strand cDNA synthesis and coupled to second-generation sequencing using the Roche GS FLX series of platforms, and is demonstrated on five SVDV isolates originating from Hong Kong during the 1970s. Sequence authenticity was validated in a multi-tiered approach (see Figure 1). Firstly through one of two criteria; either a match between a *de novo* assembly and a reference mapping, or a match between all of five different reference mappings performed against a fixed set of starting reference genomes with significant genetic distances within the same species of viruses. All reference mappings used an iterative method to avoid bias. Secondly, final validation was achieved through phylogenetic analysis against published SVDV sequences and additional *Enterovirus B* viruses using the VP2-VP3-VP1 genome region. The success of the method suggests it will be a useful tool for future analyses into the origin and spread of SVDV.

## Methods

### Virus isolates selected

Five SVDV isolates originating from Hong Kong and isolated during the 1970s (HKN/8/73, HKN/18/74, HKN/25/75, HKN/1/77 and HKN/5/77), for which the VP1 region has been Sanger sequenced previously [9], were selected (see Table 1) for near-complete genome sequencing on the Roche GS FLX system. All isolates have been held at the Pirbright Institute (UK), and grown in a pig kidney cell line (IB-RS-2) [11], since original collection as a result of past veterinary investigation into SVDV infected livestock.

**Table 1:** Results of assemblies and mappings of five Hong Kong swine vesicular disease virus (SVDV) isolates.

SVDV isolate	HKN/8/73	HKN/18/74	HKN/25/75	HKN/1/77	HKN/5/77
**Passage history**	IB-RS-2 passage 1	IB-RS-2 passage 1	IB-RS-2 passage 1	IB-RS-2 passage 2	IB-RS-2 passage 2
**Harvest date**	13May1975	29Oct1974	24March1975	7March1977	7March1977
**GenBank acc.no.**	KF963276	KF963277	KF963278	KF963274	KF963275
**Total # reads**	4287	5972	7078	7095	9985
**Total # unique reads**	4003	5489	6515	6497	9190
**Unique read lengths**					
Mean	164.2	167.9	176.1	170.7	135.2
Std. dev.	111.2	113.5	116.1	119.5	94.9
Max.	526	596	525	529	526
***De novo*** ** reads assembled**					
Coverage	3217	3660	Coding region	6314	Coding region
Min.	28	32	coverage not	84	coverage not
Max.	152	174	achieved from *de*	339	achieved from *de*
Mean	75.4	89.6	*novo* assembly	153.3	*novo* assembly
Std. dev.	20.8	24.7		40.4	
**Reads mapped against UKG/27/72 (Acc. no. X54521)**					
Coverage	3981	5449	6473	6463	9157
Min.	50	67	92	83	93
Max.	172	313	448	362	330
Mean	93.5	131.4	164.0	159.6	177.6
Std. dev.	18.8	43.4	57.2	52.0	40.4
**Reads mapped against SVDV HK'70 (Acc. no. AY429470)**					
Coverage	-	-	6472	-	9153
Min.	-	-	92	-	92
Max.	-	-	448	-	330
Mean	-	-	164.0	-	177.6
Std. dev.	-	-	57.2	-	40.4
**Reads mapped against CV-B5 ‘FAULKNER’ (Acc. no. AF114383)**					
Coverage	-	-	6473	-	9150
Min.	-	-	92	-	93
Max.	-	-	448	-	330
Mean	-	-	164.0	-	177.5
Std. dev.	-	-	57.2	-	40.3
**Reads mapped against CV-B3 ‘Woodruf’ (Acc. no. CXU57056)**					
Coverage	-	-	6474	-	9151
Min.	-	-	92	-	93
Max.	-	-	448	-	330
Mean	-	-	164.0	-	177.5
Std. dev.	-	-	57.2	-	40.3
**Reads mapped against CV-B6 ‘Schmitt’ (Acc. no. AF105342)**					
Coverage	-	5446	6473	-	9151
Min.	-	67	92	-	93
Max.	-	313	448	-	330
Mean	-	131.4	164.0	-	177.5
Std. dev.	-	43.4	57.2	-	40.3
**Number of polymorphic sites across all consensus sequences**					
**(# of which caused by matching redundant calls)**					
	1 (1)	2 (0)	0 (-)	0 (-)	0 (-)

First row shows the names of the five isolates, passage history, and harvest date. Second row shows the number of reads assigned to each isolate after they were separated using MID barcodes, and the number of these that had unique sequences (i.e. with duplicates removed), which were then kept for assembly/mapping. A number of reads is also lost during de-multiplexing, however this is even smaller than the total number of duplicates (not shown). Row three shows the mean, the standard deviation and the maximum of the read lengths of the unique reads. The fourth row shows results from *de novo* assembly, including number of reads mapped and minimum, maximum, mean, and standard deviation figures for the depth of coverage of the contig produced. The following five rows show results from iterative mapping against the five selected starting reference sequences (two SVDV sequences, a coxsackievirus B5, a coxsackievirus B3, and a coxsackievirus B6 sequence). The three isolates with successful *de novo* assemblies were iteratively mapped against one or two of these sequences for validation (see Figure 1), whereas the remaining two isolates were iteratively mapped against all five starting references. For each of these two isolates the final mapping statistics can be seen to be virtually identical across all five starting references, clearly indicating that convergence of the iterative mapping process has been obtained. The final row shows the total number of ‘polymorphic’ sites obtained when the consensus sequence from assembly (if successful) and all of the mappings are multiple-aligned for each isolate (in parenthesis is shown whether any of the ‘polymorphisms’ are due to base calls where a non-redundant call in one sequence matches the category of a redundant call in the other).

### RNA isolation, first-strand cDNA synthesis and PCR amplification

RNA was extracted using an RNeasy kit (Qiagen, Valencia, CA, USA) followed by 1^st^ strand cDNA synthesis using SuperScript III Reverse Transcriptase, RNaseOUT, and dNTPs (all Invitrogen, Carlsbad, CA, USA). All components were mixed and centrifuged briefly before use. The following reagents were mixed in a 0.2mL PCR tube at a total of 12 µL: 5 µL RNA, 2 µM oligo(dT) primer, and 0.4 mM dNTPs. Samples were incubated 5 minutes at 65°C followed by a snap-chill on ice. To the RNA/primer/dNTP mix the following reagents were added to a total of 20 µL: 1× RT buffer, 10 mM DTT, 40 U RNaseOUT, and 200 U SuperScript III RT enzyme. After a gentle mix and brief centrifugation samples were incubated 50 min at 50°C followed by 15 min at 70°C. To each sample, 2 U RNase H was added, incubated for 20 min at 37°C, and transferred to ice. PCR amplification was performed using a single primer set, which was designed to amplify all coxsackie B viruses independent of serotype and based on an alignment of all available full genome coxsackie B virus sequences in GenBank (data not shown). PCR set up was performed as previously published with forward primer: 5′-GGTGCGAAGAGTCTATTGAGC-3′ and reverse primer: 5′-CACCGAAYGCGGAKAATTTACCCC-3′ [12].

### Sample fragmentation and preparation for export from the Pirbright Laboratory

DNA concentration was quantified using a ND-1000 spectrophotometer (NanoDrop Technologies, Thermo Scientific, Wilmington, DE) prior to fragmentation using NEBNext dsDNA Fragmentase (New England Biolabs, Ipswich, MA, USA). Fragmentation was performed as follows: 3 µg DNA from each sample was added to 1× Fragmentase Reaction buffer, 1× BSA, and nuclease-free water ad hoc to 54 µL. The reaction mix was subsequently vortexed thoroughly and incubated on ice for 5 min. Three units of dsDNA Fragmentase were added to the reaction and incubated 15 min at 37°C in order to generate fragments sizes of 600–800 bp. The incubation was stopped by addition of 5 µL 0.5 M EDTA. Samples were purified using Qiagen's PCR purification kit (Qiagen, Valencia, CA, USA) according to manufacturer's guidelines and eluted in 30 uL EB buffer.

Correct fragment sizes were verified on the Agilent 2100 Bioanalyzer (Agilent Technologies, Santa Clara, CA, USA) using a DNA7500 chip. Prior to sample export, 1/10 volume of sodium acetate (3 M) was added as well as 2.5× volumes of absolute ethanol (calculated after addition of sodium acetate). Samples were incubated for 2 hours at 56°C and the tubes washed with disinfectant (FAM diluted 1/100).

### Ethanol precipitation

Amplicons from Pirbright containing sodium acetate and absolute ethanol were centrifuged at 14,000 *g* for 1 hour at 4°C. Supernatant was carefully removed and discarded, leaving a DNA pellet. Pellets were dissolved and rinsed in 150 µL ice-cold ethanol (70%) and afterwards centrifuged again for 15 mins. Supernatant was discarded and pellet was dried for 10 min at 65°C before being dissolved in 85 µL EB buffer.

### High-throughput deep sequencing

Independent sequencing libraries were produced on each sample using New England Biolabs' NEBNext DNA Sample Prep Master Mix Set 2. Samples were subsequently pooled and sequenced as just under half of 1/8 of a FLX 454 Titanium plate.

### Processing of data and sequence assembly/mapping

Sequence reads and their corresponding quality scores were imported and merged into Geneious (v.6.0.3 created by Biomatters, available from http://www.geneious.com). Reads were separated by Multiplex Identifier (MID) barcodes allowing no mismatches. Further, only unique reads were kept (see Table 1 for the number of total and unique reads for each sample following separation by barcode).

For the sake of validation and to avoid bias being introduced into the mapping process by the choice of reference sequence, a multi-tiered approach was chosen to obtain consensus sequences, and is illustrated in detail in Figure 1. For each sample *de novo* assembly was attempted using the Geneious assembler (v.6.0.3) [13] starting with the lowest standard sensitivity setting, and if this failed to deliver a full-length contig the sensitivity was incremented following the standard settings. If a full-length contig was obtained, the veracity of the *de novo* consensus sequence was substantiated by performing an iterative mapping against one or more starting reference sequences (the starting reference sequence is only used in the first round in the iterative mapping process, whereas all subsequent rounds use the consensus from the contig of the previous round as reference), and ensuring that the two consensus sequences matched (see Table 1 and Figure 1). If no full-length contigs were obtained in the *de novo* assembly, multiple iterative mappings against starting references of varying genetic distance (within the same species but including different serotypes, see Table 1) were performed using the Geneious Read Mapper (v.6.0.3) (see also http://assets.geneious.com/documentation/geneious/GeneiousReadMapper.pdf). The consensus sequences of these were then checked for identity and upon confirmation of this, the sequence was accepted. Mappings to (starting) references, including those used to confirm *de novo* results, used the medium sensitivity setting in Geneious (v.6.0.3) and importantly all used the iterative mapping feature, ensuring that sufficient iterations were allowed for mappings to converge to a stable (and thus unbiased) consensus sequence. All consensus sequences were called using a 50% strict consensus criterion. For samples where there were any differences between *de novo* assembly and reference mapping consensus sequences, the *de novo* derived result was chosen as final.

### Phylogenetic-based sequence verification

We further validated the sequence assemblies through a phylogenetic approach. This was primarily done to ensure that the serology and sample provenance made sense in light of cladistics. Due to what is known of the nature of recombination in this genus of viruses, and because it is what determines serology, as it forms the external part of the capsid, the VP2-VP3-VP1 genome region was chosen for analysis. In addition to the five samples, publically available sequences from 50 isolates of SVDV and Coxsackievirus B1 to B6 were aligned in the MAFFT [14] plugin (v.1.3) in Geneious (v.6.0.3), and visually inspected. After manual removal of gaps and ambiguous regions the resulting alignment of 2,286 nucleotides in length was used for subsequent analyses. The molecular model of substitution was selected by testing in jModeltest v. 2.1.1 [15] using four Gamma categories. Both the Akaike information criterion [16] and the corrected Akaike information criterion [17] chose the GTR + I + G model as the best, and this was used during the subsequent maximum likelihood based phylogenetic analysis in the PhyML [18] plugin (v.2.2.0) in Geneious (v.6.0.3) using 100 nonparametric bootstrap pseudoreplicates as support. The tree was visualized in FigTree v.1.4.0 (Rambaut A, FigTree v.1.4.0. Available at http://tree.bio.ed.ac.uk/software/figtree, 2013).

## Results

### Complete protein-encoding genomes

Nucleotide sequences encompassing the complete 6,558 bases coding region of five Hong Kong SVDV isolates (all from the 1970s) were deep sequenced and assembled into a 6,938 nucleotide sequence, extending 307 bases upstream of the coding region and 73 bases downstream (excluding stop codon). The sequences have been deposited in NCBI's GenBank under reference accessions KF963274- KF963278. Further, sequence data are accessible through EBI's European Nucleotide Archive with study accession number PRJEB5810 (for direct access: http://www.ebi.ac.uk/ena/data/view/PRJEB5810).

Assembly and mapping statistics are given in Table 1. The strong similarity for the statistics between mappings against different starting references for the same sample is a sign of the strength of the method, as they represent the statistics of the final resulting contig of the iterative mapping process. Thus they are a reflection of the fact that the mappings have all converged. This is further reflected in the lack of difference in the consensus sequences. For the samples where *de novo* was successful at full-length assembly, there are very few differences between the consensus sequence from these and those from the mappings (see Table 1) with a maximum of two polymorphic sites between all assembly/mappings for any sample. This level of accord should serve as a guide when applying the current methodology.

### Phylogenetic reconstruction using a Maximum Likelihood approach

**Figure 2:**
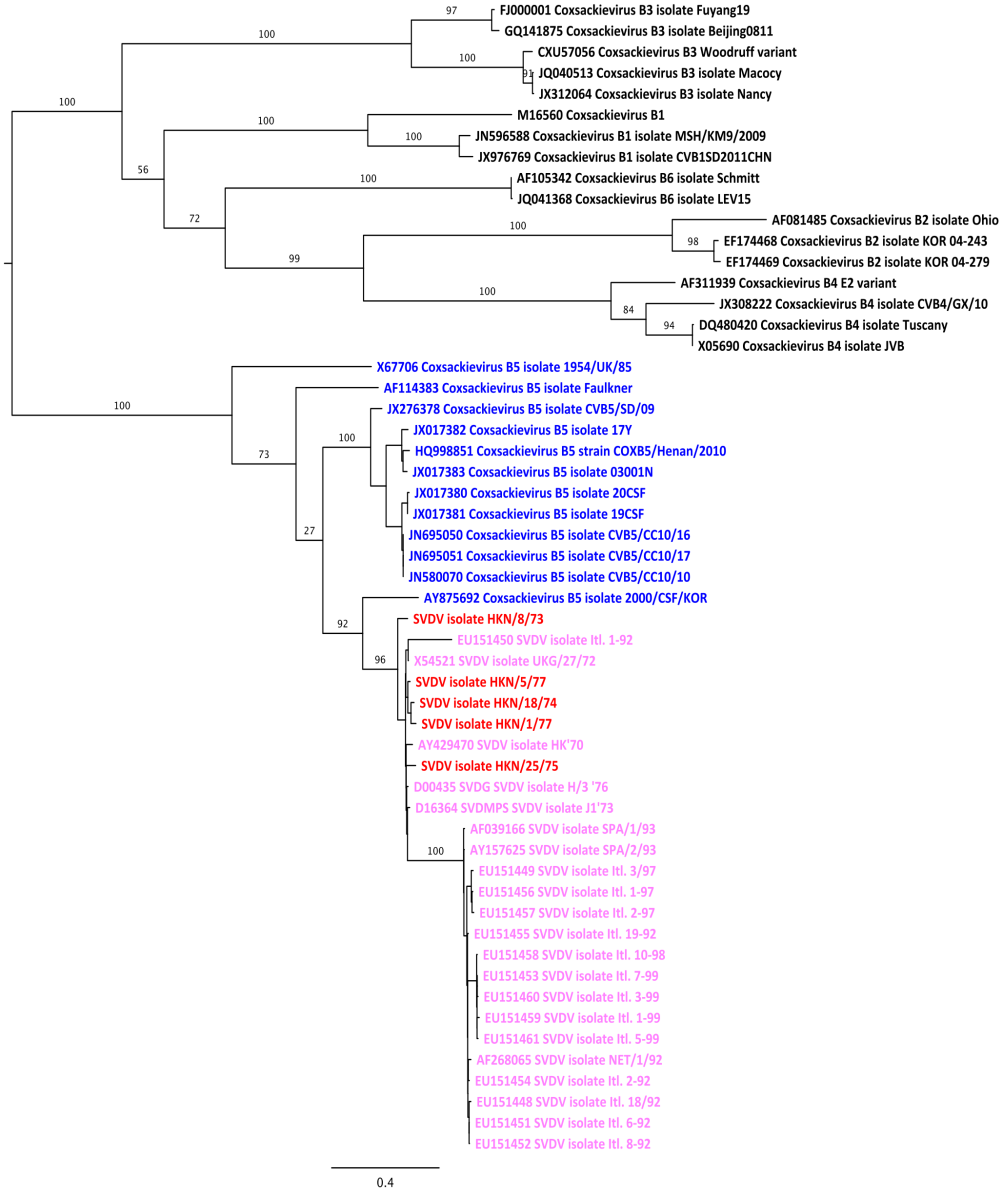
Phylogenetic validation of samples. Using a maximum likelihood approach as described in Methods on the 1B-1C-1D genome region, corresponding to the outer capsid proteins VP2, VP3 and VP1, final validation was obtained for the samples. The five Hong Kong SVDVs isolated in the 1970s and which were sequenced in this study, are shown in red and all other SVD virus isolates are shown in pink. Coxsackievirus B5 isolates are shown in blue, and other *Enterovirus B* serotypes are shown in black. As expected from the previous literature, all CV-B5 together with all SVDV form a monophyletic cluster. This is supported with a bootstrap proportion of 100/100. Within this cluster all the SVD virus isolates, including those sequenced in this study, form a monophyletic cluster with bootstrap support of 96/100. Additionally, none of the five presently sequenced Hong Kong isolates show any obvious aberrations with regards to their position in the topology concerning either geographical information or branch lengths (vs. age). The tree is rooted on the branch leading from CV-B5 to all other CV-B sequences, and all branch labels show support in the form of bootstrap proportions out of one hundred. Support values for nodes with minor importance regarding the verification are not shown.

At nearly three times the length of alignments typically employed for VP1 based phylogenies, our alignment can be considered highly informative. The phylogenetic tree (Figure 2), rooted on the branch between the CV-B5's and all other CV-B's, shows all CV-B5 sequences and all SVDV sequences forming a highly supported (bootstrap proportion 100/100) monophyletic cluster, with the SVDV sequences themselves forming a highly supported (bootstrap proportion 96/100) monophyletic cluster within this. This conforms to the currently accepted evolutionary relationship of the strains for this genomic region. Furthermore, the topology shows no apparent aberrations with regards to the geographical location or age of isolates vs. branch lengths with regards to the five SVDV samples.

## Discussion

The principal aim of this study was to develop a simple and rapid workflow for the sequencing of near-complete SVDV genome sequences, under the assumption that the samples contain suitable quality RNA for initial first-strand cDNA synthesis. With many samples available in cultures in reference banks such as that held at the Pirbright Institute, this is not an unlikely expectation. A limitation of the method, however, is that as it is based upon a single long-range PCR reaction, it will not be suitable for samples in which the SVDV RNA molecules are not near-complete length, thus in those situations other methods should be considered. Nevertheless, for isolates were the genome is intact, the primer set presented here will allow for the rapid generation of the complete coding genome.

The aim of this study was purely methodological, thus we do not wish to dwell on the phylogenetic results. However, the analysis demonstrates that all sequences cluster, as expected, among published SVDV sequence data, and overall the phylogenetic relationship for the full dataset is consistent with that previously generated using the VP1 gene sequences by Zhang et al. [9]. The data furthermore shows that assembly and mapping of the viral genomes and use of the validation strategy employed provides unbiased consensus sequences and is thus suitable for obtaining reliable results. Furthermore, contigs and assemblies have a sufficiently high and evenly distributed coverage to make them suitable for intra-host studies, should this be desired.

### Concluding remarks

In this study, near-complete genomes, encompassing complete coding regions, of five cultured SVDV isolates were generated in a simple and rapid manner. Given the known existence of large numbers of similarly cultured samples in archives, and known questions about the time and source of origin of SVDV as a livestock pathogen, we anticipate that our method may be of use for future studies that wish to address this question in more detail.
